# Structure of Antimicrobial Stewardship Programs in Leading US Hospitals: Findings of a Nationwide Survey

**DOI:** 10.1093/ofid/ofz104

**Published:** 2019-04-06

**Authors:** Derrick Nhan, Eric J M Lentz, Marilyn Steinberg, Chaim M Bell, Andrew M Morris

**Affiliations:** 1 University of Toronto, Toronto, ON, Canada; 2 Department of Medicine, McMaster University, Hamilton, ON, Canada; 3 Sinai Health System, Toronto, ON, Canada; 4 University Health Network, Toronto, ON, Canada; 5 Department of Medicine, University of Toronto, Toronto, ON, Canada

**Keywords:** antibiotic resistance, antimicrobial stewardship, antimicrobial use

## Abstract

**Background:**

To examine antibiotic stewardship program (ASP) structure among high-performing hospitals and determine which components of the 2016 Infectious Diseases Society of America (IDSA)/Society for Hospital Epidemiology of America (SHEA) ASP guidelines are implemented at each site.

**Methods:**

A survey of the highest-ranking hospitals, compiled from the 2015–2016 US News and World Report’s Best Hospital Rankings, was conducted from August to December 2016. This corresponded to 138 adult and 62 pediatric unique hospitals. We inquired as to which components of the 2016 IDSA/SHEA ASP guidelines were implemented at each site. Appropriate persons at each hospital were emailed surveys after telephone or email conversations confirmed that they belonged to that hospital’s ASP.

**Results:**

Overall, 101 of 200 hospitals responded (51%). Of these, 82% (n = 83/101) had an active ASP, and 59% (n = 47/80) were active for more than 5 years. Most report to a committee rather than to an individual (n = 68/80, 85%), do not have their own budget (n = 42/80, 53%), and selectively implement IDSA/SHEA recommendations. Additionally, the majority of ASPs in top hospitals follow aspects of The Joint Commission Standards for Antimicrobial Stewardship, which were released after the survey was administered.

**Conclusions:**

Of leading US hospitals responding to our survey, >80% had an ASP, and most implemented the majority of commitments, interventions, and optimization strategies suggested by IDSA/SHEA. Understanding the structure of ASPs in these hospitals will assist other hospitals in program implementation.

Antibiotics have changed the course of medicine, but their overuse has contributed to the emergence of antibiotic-resistant bacteria [1]. For instance, in 2013, the Centers for Disease Control and Prevention (CDC) estimated that antibiotic-resistant bacteria are responsible for >2 million infections and 23 000 deaths each year [2]. Furthermore, the health care costs of antibiotic-resistant infections have been estimated to be higher than $20 billion annually [3].

One strategy to counter antibiotic resistance is the implementation of antibiotic stewardship programs (ASPs) [4]. Generally, an ASP is responsible for promoting the appropriate use of antibiotics [5]. The profile of ASPs has grown, with the US government calling for the establishment of ASPs in all acute care hospitals by 2020 in the National Action Plan for Combating Antibiotic-Resistant Bacteria, published in 2015 [6], supported by a similar call by the US Centers for Disease Control in 2014 [2], standards set by The Joint Commission in 2017 [7], and standards proposed by the Centers for Medicare and Medicaid Services in 2016 [8].

Although all hospitals are expected to have ASPs by 2020, we theorized that top-ranked ones would be more advanced in their ASP adoption and practices and may help outline future directions for the field. Thus, we surveyed the top 138 adult and top 62 pediatric hospitals in the United States, as determined by US News and World Report’s Best Hospital Rankings, to determine the existence and characteristics of ASPs at these institutions [9]. The survey was conducted in US hospitals, as there are no guidelines in Canada comparable to the Infectious Diseases Society of America (IDSA)/Society for Hospital Epidemiology of America (SHEA) guidelines.

## METHODS

The 2016 IDSA/SHEA guidelines set forth a framework of evidence-based recommendations for implementing an effective institutional ASP [10]. We developed a survey based on these guidelines to examine which of these recommendations were implemented in top US hospitals and also addressed additional subjects, such as the different roles of ASP team members, the structure of the ASP, and support provided by hospital administration. This survey was done to gain a better understanding of the current landscape of ASPs. To generate our list, the 50 highest-ranking hospitals from each specialty of the 2015–2016 US News list of top hospitals was compiled. As shown by Wang et al., there seems to be correlation with regards to hospital performance when comparing top-ranked US News hospitals vs unranked institutions [11]. The criterion used by US News to rank the majority of these institutions was the Index of Hospital Quality score, based on 4 weight-based components: outcomes (32.5%), structure (30.0%), process (27.5%), and patient safety (10.0%). Four specialties (ophthalmology, psychiatry, rehabilitation, and rheumatology) were given rankings based on reputation only. Any duplicate adult or pediatric hospitals on the US News list were subsequently deleted. The study and survey tool received approval from our institutional Research Ethics Board.

Recruitment phone calls to hospitals were made between August and November 2016. Contact information for ASPs and other departments or people who may have been involved with each hospital was collected from their respective websites. If obtainable, an email was sent when potential contacts could not be reached by phone. A follow-up email was sent 2 weeks after the initial email if there was an incomplete or no response. When no information was available online, the switchboard was used to reach the inpatient pharmacy, the Infection Prevention and Control Program, or the Infectious Disease Department to speak to an ASP member or obtain ASP contact information.

The online survey was generated using SurveyMonkey. Links to the survey were included in all emails. Once the collection period ended, the Internet Protocol (IP) addresses of all responses were compared; if there were found to be multiple responses with the same IP address, the most complete entry was kept, with the more incomplete ones being deleted. These incomplete entries were likely from hospitals starting multiple survey entries due to being unable to pick up where they had left off. Additionally, 16 of the adult and pediatric hospitals overlapped and shared the same ASP; these hospitals were only counted in the adult list. We also found during the processing of the data that affiliated hospitals appearing as unique entries on our list actually shared an ASP. In these cases, only the most complete entry, arbitrarily followed by the earliest survey entry, was counted, and both hospitals were treated as 1, with the total being adjusted accordingly. We used descriptive statistics to present results.

The Joint Commission published a series of medication management standards relevant to antimicrobial stewardship that became effective on January 1, 2017, describing the standards that ASPs must meet to be accredited [9]. In addition to comparing our results with the guidelines set forth by the IDSA/SHEA about implementing effective ASPs, we were able to use our results to gain insight into the existence of certain Joint Commission standards in the ASPs surveyed.

## RESULTS

The final adjusted number of hospitals was 138 adult and 62 pediatric. Overall, 101 of 200 hospitals (51%) responded. For adult hospitals, 69 of 138 (50%) responded, 23 of 62 (37%) pediatric hospitals responded, and 9 responses were incomplete but were still used in the final count. We were unable to determine whether these incomplete responses came from pediatric or adult hospitals; using their IP addresses, however, we were able to verify that they were unique. Many respondents chose not to answer every question.

Table 1 details several structural elements of the ASPs that were surveyed. Of the hospitals that responded, 82% (n = 83/101) had an active ASP program. Fifty-nine percent (59%, n = 47/80) of them had been implemented for 5+ years, 19% (n = 15/80) for 3–5 years, 18% (n = 14/80) for 1–3 years, and 5% (n = 4/80) for <1 year. Most ASPs (73%, n = 58/80) operated within a program or department. ASPs largely reported to committees (85%, n = 68/80); a smaller percentage reported to an individual (10%, n = 8/80). Slightly less than half of the hospitals surveyed reported having a budget solely dedicated to the ASP (48%, n = 38/80). Funding for hospitals with a dedicated budget came primarily from the Department of Pharmacy (61%, n = 22/36) or via joint contribution between 2 or more departments, most commonly the Departments of Pharmacy and Infectious Diseases (31%, n = 11/36) (Table 1). Of these budgets, most were between $50 000 and $150 000 (32%, n = 7/22) or $151 000 and $250 000 (32%, n = 7/22) annually.

**Table 1.** Survey Responses Relating to the Structure and Organization of Antimicrobial Stewardship Programs in Top-Ranking American Hospitals^a^

Does your institution have an active ASP? (n = 101)	No. of Respondents	%
Yes	83	82
No	18	18
How long has the ASP been active as of July 1st, 2016? (n = 80)		
<1 y	4	5
1–3 y	14	18
3–5 y	15	19
>5 y	47	59
Who are the leaders of the ASP at your facility? (n = 78)		
Physician and pharmacist co-leads	46	59
Physician	27	35
Pharmacist	5	6
Does your ASP fall within a program/department? (n = 80)		
Yes	58	73
No	22	28
What hospital program or department does your ASP operate within? (n = 61)		
Pharmacy	35	58
Both Infectious Disease and Pharmacy	19	31
Infectious Disease	2	3
Quality and Safety	3	5
Other	2	3
Does your ASP report to a committee or individual? (n = 80)		
Committee	68	85
Individual	8	10
Neither	4	5
If your ASP reports to a committee, what committee does it report to? (n = 68)		
Pharmacy and Therapeutics	43	63
Infection Disease/Control	6	9
Quality Control/Improvement	4	6
Medical Executive Committee	2	3
More than 1 of the above committees	13	19
If your ASP reports to an individual, what is their title? (n = 8)		
Chief Medical Officer	3	38
Leader of Quality Affairs/Chief Quality Officer	2	25
Chief Nursing Officer	1	13
VP of Medical Affairs	1	13
Chief Safety Officer	1	13
Is there a budget dedicated solely to the ASP? (n = 80)		
No	42	53
Yes	38	48
Under which program or department does the ASP budget fall? (n = 36)		
Pharmacy	22	61
Infectious Disease	1	3
Health Care Quality	2	6
More than 1 of the above groups	11	31
In thousands of dollars, approximately how much is this budget annually? (n = 22)		
<50	1	5
50–150	7	32
151–250	7	32
251–350	3	14
351–450	4	18
What are the “other” roles in your ASP (other roles = roles besides physicians, pharmacists, nurses, information technologists, and epidemiologists)? (n = 35)		
Microbiologist	22	63
Infection Prevention and Control	10	29
Other	3	9

Abbreviation: ASP, antibiotic stewardship program.

^a^Where the sample size, n, is less than expected (eg, for most questions, the eligible number of respondents was 83), it reflects that no response was provided.

**Figure 1.**
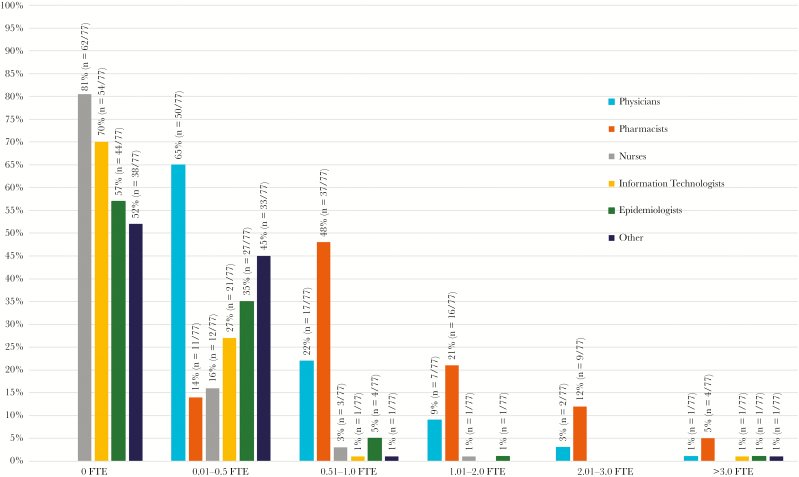
Survey responses to questions about the staff and members of antimicrobial stewardship programs in top-ranking American hospitals. Each individual question asked how many full-time equivalents (FTEs) of each role the program had and gave each of the FTE ranges listed in the figure as answer options. Seventy-seven hospitals answered each question.

Shown in Figure 1 and Table 1 are the full-time equivalent (FTE) personnel for the surveyed hospitals. All ASPs had physicians and pharmacists as part of their team. With regard to ASP structure, 65% (n = 50/77) had ≤0.5 FTE physicians, and 22% (n = 17/77) had 0.51–1.0 FTE physicians. For pharmacists, 48% (n = 37/77) of programs had 0.51–1.0 FTE pharmacists, and 21% (n = 16/77) had 1.01–2.0 FTE pharmacists.

Table 2 details many of the policies and practices of surveyed ASPs. Institutional commitment to ASPs included salary support (90%, n = 71/79), ensuring participation from relevant departments (85%, n = 67/79), support for training and education regarding stewardship (80%, n = 63/79), providing staff adequate time to take part in ASP activities (75%, n = 59/79), a formal statement by the facility to improve antibiotic use (72%, n = 57/79), and the inclusion of stewardship duties in job descriptions and performance reviews (67%, n = 53/79). The most common interventions utilized by ASPs to reduce inappropriate antimicrobial usage incorporated prospective feedback and audit (88%, n = 65/74), facility-specific recommendations for infectious syndromes (88%, n = 65/74), and antimicrobial pre-authorization (82%, n = 61/74). About three-quarters (77%, n = 57/74) of ASPs had a system in place to monitor adherence to antimicrobial recommendations following feedback to the prescriber. The most common ASP optimization strategies included promoting intravenous (IV) to oral antimicrobial transition where appropriate (93%, n = 68/73). Only 4 of the ASPs surveyed implemented all IDSA/SHEA recommendations regarding reducing inappropriate use of antibiotics, and only 7 implemented all recommendations for optimizing antimicrobial optimization strategies. Most commonly, success in reducing inappropriate antimicrobials was measured using days of therapy (DOTs; 92%, n = 67/73), *Clostridium difficile* infection rates (89%, n = 65/73), and measures of antimicrobial resistance (75%, n = 55/73). With respect to antimicrobial expenditures, 82% (n = 55/67) of ASPs utilized purchasing data, whereas 40% (n = 27/67) used prescribing/administering costs. There was no consistent pattern as to how often antimicrobial prescribing and resistance patterns were reported to staff. Clinician education commonly took the form of didactic lectures (88%, n = 61/69).

**Table 2.** Survey Responses to Questions About Antimicrobial Stewardship Program Policies and Practices in Top-Ranking American Hospitals

At your hospital, is the commitment to your ASP in the form of: (n = 79)	No. of Respondents	%
Salary support for ASP members	71	90
Ensuring participation from departments able to support the antimicrobial stewardship program	67	85
Support for training and education regarding antimicrobial stewardship	63	80
Allowing staff from relevant departments adequate time to participate in antimicrobial stewardship activities	59	75
A formal statement by the facility supporting efforts to improve antimicrobial usage	57	72
The inclusion of stewardship duties in job descriptions and performance reviews	53	67
Which of the following interventions to reduce inappropriate antimicrobial use does your ASP utilize? (n = 74)		
Prospective audit and feedback of antimicrobial prescriptions for the prescriber	65	88
Facility-specific recommendations for specific infectious syndromes	65	88
Pre-authorization/formulary restrictions of antimicrobials	61	82
Antimicrobials restricted to ID physicians	52	70
Facility-specific recommendations for common infectious syndromes	50	68
Antimicrobial stewardship program intervention in cases with high risk of *Clostridium difficile*	27	36
Antimicrobial time-outs	17	23
Is there any system in place to monitor adherence to antimicrobial recommendations following feedback to the prescriber? (n = 74)		
Yes	57	77
No	17	23
Which of the following antimicrobial optimization strategies does your ASP utilize? (n = 73)		
Promoting the use of and transition toward oral antimicrobials over IV antimicrobials where appropriate	68	93
Strategies to minimize duration of antimicrobial therapy	56	77
Dedicated pharmacokinetic monitoring and adjustment program for patients on IV aminoglycosides	50	68
Documentation of dosing, duration, and indication for antimicrobials	43	59
Use of a computerized clinical decision support system when prescribing antimicrobials	36	50
Time-sensitive stop orders	27	37
Which of the following measurements of impact on antimicrobial use does your ASP utilize? (n = 73)		
Days of therapy	67	92
Rate of *Clostridium difficile* infection	65	89
Measures of antimicrobial resistance	55	75
Defined daily doses	55	75
Use of key clinical outcomes for specific infectious syndromes	9	12
Which of the following measurements of impact on antimicrobial expenditure does your ASP utilize? (n = 67)		
Purchasing data	55	82
Prescribing/administering costs	27	40
How often does your ASP report antimicrobial prescribing and resistance patterns to relevant staff? (n = 74)		
Every 1–3 mo	15	20
Every 4–6 mo	13	17
Every 7–12 mo	30	40
Never	13	17
Other	5	7
Which of the following strategies (if any) does your ASP use to educate clinicians regarding resistance and optimal prescribing habits? (n = 69)		
Didactic lectures/presentations	61	88
Web-based educational resources	30	43
Education pamphlets	13	19
Posters/flyers	13	19
Reviewing de-identified cases with providers	8	12
Other	8	12
None	1	1

Abbreviations: ASP, antibiotic stewardship program; ID, infectious disease; IV, intravenous.

## DISCUSSION

We surveyed the top-ranking acute care adult and pediatric hospitals in the United States to provide insight into the prevalence, structure, and policies of ASPs and compared this with the recommendations made by the Infectious Diseases Society of America and the Society for Healthcare Epidemiology of America, and with The Joint Commission standards for Antimicrobial Stewardship. We found that 82% of hospitals had an ASP, and most implemented the majority of commitments, interventions, and optimization strategies suggested by the IDSA/SHEA [10]. Specifically, 5% (n = 4/74) of hospitals implemented all recommended interventions to reduce inappropriate use (eg, “Prospective audit and feedback of antimicrobial prescriptions for the prescriber”), and 10% (n = 7/73) of institutions implemented all optimization strategies (eg, “Promoting the use of and transition toward oral antimicrobials over IV antimicrobials where appropriate”) (Table 2). Most surveyed ASPs followed The Joint Commission standards that became effective after the survey on January 1, 2017 [12].

Compared with the international and North American averages of 58% and 67%, respectively, found in a 2018 survey, 82% of hospitals that responded to our survey had an active ASP [13]. Surveys of hospitals in China, the Netherlands, and Ontario, Canada, found 94.8%, 94%, and 88% of responding hospitals to have ASPs, respectively [14–16]. The international survey included hospitals from many countries that are less developed and do not require the presence of ASPs in medical institutions, whereas in China, the Netherlands, and Ontario, ASPs have been required since 2011, 2014, and 2013, respectively [14–16]. We therefore anticipated that the prevalence of ASPs among the institutions we surveyed would be lower than that found in other surveys.

In a similar 2013 survey conducted in hospitals that were part of the Yankee Alliance and Premier Healthcare Alliance in the United States, 51% of respondents reported having an ASP [17]. Another 2013 national survey conducted by the American Society of Health-System Pharmacists found that 63% of participating hospitals had ASPs [18]. Hospitals on our list, in comparison, were likely to be larger, urban, and have better access to resources to set up an ASP. As sufficient funding and staffing are major barriers to the implementation of a formal ASP, the difference between these studies and our own is expected [13, 17, 19]. Additionally, urban hospitals are more likely than their rural counterparts to have an ASP [20].

The 2013 Yankee Alliance-Premier Healthcare Alliance survey also found that 54% of ASPs had existed for at least 1 year, compared with 96% in our study [17]. This may indicate a rapid expansion in formal hospital ASPs over a period of over 3 years, although it may also reflect differences in hospital resources between the 2 study cohorts. A study from the 2014 National Healthcare Safety Network (NHSN) Annual Hospital Survey found that 53% of hospitals expressed commitment to their ASP through a written statement of support, whereas 32% achieved this through ASP staff salary support [21]. In comparison, our survey found that support for each of the aforementioned was 72% and 90%, respectively. In contrast to our results, the NHSN also found that ASPs are more likely to be led by pharmacists than physicians [18]. The frequencies of interventions implemented by ASPs, such as specific recommendations and audit and feedback, were similar in both studies.

Of hospitals with an ASP, most reported that the ASP had been active for more than 5 years (59%), indicating ample time to develop and optimize their structure and practices. Despite this, less than half of ASPs surveyed had a dedicated budget (48%). Of programs that responded, budgets ranged from less than $50 000 to between $350 000 and $450 000, with 64% of these budgets falling in the $50 000 to $250 000 range. Although not explored in our survey, a study of pediatric hospitals in 2016 revealed that the cost of ASPs varied from $17 000 to $388 500 annually and did not seem to correlate with the size of the hospital [22]. This highlights inconsistent hospital ASP funding. Furthermore, considering that studies have shown that ASPs have a significant impact on reducing hospital stay and expenditure, it would seem that there is ample justification for a dedicated budget [23–26]. Nowak et al. examined economic outcomes of the ASP at Wake Forest Baptist Medical Center and estimated yearly drug cost savings ranging from $920 070 to $2 064 441 [25]. Drew et al. noted that ASPs, in both the community and academic settings, reduced antimicrobial usage by 22%–36% and saved approximately $200 000–$900 000 in annual drug expenditures [26].

Of ASPs surveyed that responded, most reported to a committee (85%), usually Pharmacy and Therapeutics, rather than to an individual. This can limit the program’s success because of poor alignment with hospital financial support and a diffusion of responsibility and accountability.

The least common intervention to reduce inappropriate antimicrobial use among surveyed hospitals was antimicrobial time-outs (23%), and the least common optimization strategy was time-sensitive stop orders (37%). A similar study by Poteete et al. regarding implemented ASP interventions also found that time-sensitive stop orders were the least utilized [27]. These findings demonstrate that even the top ASPs do not implement all the recommended interventions consistently.

Ninety-two percent (92%) of hospitals responding to our surveyed reported using DOTs as a way to measure impact on antimicrobial use, and 75% reported using DDDs. In contrast to this, hospitals with ASP programs in China, the Netherlands, and Ontario used DDDs more frequently than DOTs, where 96%, 81%, and 52% of hospitals reported using DDDs, but only 70%, 29%, and 39% reported using DOTs, respectively [14–16]. Eighty percent of hospitals surveyed internationally reported using DDDs [13]. The international survey did not ask about measurement of DOTs.

Our survey addresses 6 of the 8 Joint Commission standards for hospital ASP accreditation in some capacity [12]. The first standard notes that the establishment of an antimicrobial stewardship program should be prioritized in hospitals. At the time of our survey, almost one-fifth (18%) of top hospitals did not have an active ASP, falling short of this standard. The second relevant standard involves educating hospital staff and practitioners about antimicrobial stewardship practices. This was accomplished by almost all hospitals with an ASP program in our study. Another standard relates to the membership in an ASP. Although all programs with ASPs included physicians and pharmacists as core members, only about one-third included microbiologists, and even fewer included an infection preventionist and/or a nurse. The importance of a multidisciplinary approach, including microbiology, infection prevention and control, and nursing in creating successful ASPs has been noted [28, 29]. Top medical hospitals have room to improve on standards related to the collection of data and implementation of policies and procedures related to antimicrobial use and management. For example, roughly one-quarter (23%) of the ASPs surveyed implement antimicrobial time-outs as a method to reduce inappropriate antimicrobial use, a recommended action referred to in The Joint Commission document [12]. Lastly, the standards list a number of core elements that ASPs should have, including leadership, accountability, drug expertise, action, tracking, reporting, and education. Our survey was unable to formally assess all elements but does show that most ASPs meet the action, tracking, reporting, and education recommendations.

This study has some limitations. First, the response rate of just over 50% may introduce nonresponse bias and affect generalizability. Many hospitals did not respond to requests to participate in the survey after multiple attempts to contact them, whereas others responded that it was their policy to not participate in such studies. Several respondents also did not fully complete the survey. We might expect lower than observed estimates from the nonresponders, indicating that there is still room to improve with ASPs in hospitals. Second, there was no way to verify if the information provided by respondents was accurate. For example, a social desirability bias may have caused some hospitals to overstate certain features of their ASPs. Third, it may be the case that hospitals with an ASP or hospitals that are more passionate about such activities were more likely to respond to our survey requests. This could inflate the data collected for the prevalence of ASPs among top-ranking US hospitals and introduce further bias to other questions. This would also make the data collected for hospitals with an ASP more representative of leading ASPs, as, presumably, hospitals that are more passionate about ASP activities have more developed programs. Additionally, it is also possible that respondents misunderstood our question on budgets, as there was no attempt to differentiate cost from budget. Lastly, our survey does not capture information about the intensity or details of the interventions and strategies surveyed.

Our study found that more than three-quarters of the top-ranked hospitals in the United States responding to our survey have a formal antimicrobial stewardship program. Although many of the antimicrobial stewardship programs adhere to recommended practices from the IDSA/SHEA, only 5% (n = 4/74) implemented all recommended interventions to reduce inappropriate use, and 10% (n = 7/73) implemented all optimization strategies. Thus, our findings demonstrate that there is room to improve, even in top hospitals. Enhancements in governance, organizational structure, and accountability can help enhance program effectiveness, administrative efficiency, and focus. Given the high number of deaths caused annually by antimicrobial-resistant organisms and the great financial burden antimicrobial resistance places upon health care systems, it is crucial to understand the structure of ASPs in hospitals so that we are able to effectively tackle the growing problem of antimicrobial resistance [30].
